# Modulation of hippocampal astrocytes in aged rats through insulin‐growth factor 1 gene therapy

**DOI:** 10.1002/alz70855_104067

**Published:** 2025-12-23

**Authors:** Ana Abril Vidal Escobedo, Facundo Peralta, Julia Emilia Alejandra Diaz Baliero, Joaquín Pardo, Paula Cecilia Reggiani

**Affiliations:** ^1^ National Council of Scientific and Technical Research (CONICET/UNLP), La Plata, Buenos Aires, Argentina; ^2^ National Council of Scientific and Technical Research (CONICET/UNLP), La Plata, Argentina

## Abstract

**Background:**

Aging is associated with an increased incidence of neurodegenerative diseases and neuroinflammation in the hippocampus, a key brain region involved in memory. Microglia and astrocytes play critical roles in these processes: microglia initiate immune responses, while astrocytes regulate inflammation and maintain homeostasis. In aged brains, microglia are often become chronically reactive, which may contribute to cognitive decline. Concurrently, astrocytes in the hippocampus become fewer, smaller, and exhibit less complex branching. A promising therapeutic strategy involves the Insulin‐like Growth Factor 1 (IGF1), a neuroprotective molecule shown to enhance memory, promote neurogenesis, and exert anti‐inflammatory effects. Building upon our previous work using an adeno‐associated viral (AAV) vector to overexpress IGF1 in hippocampal astrocytes, we now aim to explore its effects on age‐related neurodegeneration, specifically focusing on behavioural and molecular outcomes.

**Method:**

Twenty‐month‐old female Sprague Dawley rats were divided into Intact and IGF1. On day 0, intrahippocampal injections of an AAV vector carrying the gfaABC1D promoter (specific for astrocytes) to drive IGF1 and TdTomato expression were performed. Behavioural evaluations began on day 42 and included open field, object recognition, and Barnes maze tests. On day 52, animals were euthanized, and their brains preserved for subsequent molecular analysis. Immunohistochemical labelling was conducted to examine immature neurons (DCX) and microglia (IBA‐1).

**Result:**

The expression of TOM in the hippocampus confirmed successful transduction, with astrocytes expressing both IGF1 and TOM. Preliminary results showed that aged rats with hippocampal astrocytes overexpressing IGF1 exhibited improvements in certain behaviours, including increased rearing, enhanced long‐term object recognition, and improved spatial memory. However, molecular analyses of DCX expression did not reveal significant changes, and IBA‐1 marker have yet to be analysed.

**Conclusion:**

This study presents an innovative therapeutic strategy targeting hippocampal astrocytes in aged rats using a genetic vector to overexpress IGF1. The treatment successfully modulated IGF1 expression in these cells, resulting in partial improvements in behaviour and memory function, highlighting its potential for age‐related cognitive decline.

